# Eradication of LIG4-deficient glioblastoma cells by the combination of PARP inhibitor and alkylating agent

**DOI:** 10.18632/oncotarget.26409

**Published:** 2018-12-07

**Authors:** Monika Toma, Monika Witusik-Perkowska, Marzena Szwed, Robert Stawski, Janusz Szemraj, Malgorzata Drzewiecka, Margaret Nieborowska-Skorska, Maciej Radek, Pawel Kolasa, Ksenia Matlawska-Wasowska, Tomasz Sliwinski, Tomasz Skorski

**Affiliations:** ^1^ Laboratory of Medical Genetics, Faculty of Biology and Environmental Protection, University of Lodz, Lodz, Poland; ^2^ Department of Medical Biochemistry, Medical University of Lodz, Lodz, Poland; ^3^ Department of Medical Biophysics, University of Lodz, Lodz, Poland; ^4^ Department of Clinical Physiology, Medical University of Lodz, Lodz, Poland; ^5^ Department of Microbiology and Immunology, Fels Institute for Cancer Research and Molecular Biology, Temple University Lewis Katz School of Medicine, Philadelphia, PA, USA; ^6^ Department of Neurosurgery, Surgery of Spine and Peripheral Nerves, Medical University of Lodz, University Hospital WAM-CSW, Lodz, Poland; ^7^ Department of Neurosurgery, Medical University of Lodz, Copernicus Memorial Hospital, Lodz, Poland; ^8^ Social Sciences Academy in Lodz, Lodz, Poland; ^9^ Division of Pediatric Research, Department of Pediatrics, University of New Mexico Health Sciences Center, Albuquerque, NM, USA

**Keywords:** glioblastoma, PARP inhibitor, LIG4, alkylating agent, synthetic lethality

## Abstract

Cancer cells often accumulate spontaneous and treatment-induced DNA damage i.e. potentially lethal DNA double strand breaks (DSBs). Targeting DSB repair mechanisms with specific inhibitors could potentially sensitize cancer cells to the toxic effect of DSBs. Current treatment for glioblastoma includes tumor resection followed by radiotherapy and/or temozolomide (TMZ) – an alkylating agent inducing DNA damage. We hypothesize that combination of PARP inhibitor (PARPi) with TMZ in glioblastoma cells displaying downregulation of DSB repair genes could trigger synthetic lethality. In our study, we observed that PARP inhibitor (BMN673) was able to specifically sensitize DNA ligase 4 (LIG4)-deprived glioblastoma cells to TMZ while normal astrocytes were not affected. LIG4 downregulation resulting in low effectiveness of DNA-PK–mediated non-homologous end-joining (D-NHEJ), which in combination with BMN673 and TMZ resulted in accumulation of lethal DSBs and specific eradication of glioblastoma cells. Restoration of the LIG4 expression caused loss of sensitivity to BMN673+TMZ. In conclusion, PARP inhibitor combined with DNA damage inducing agents can be utilized in patients with glioblastoma displaying defects in D-NHEJ.

## INTRODUCTION

Glioblastoma (grade IV in WHO Classification of Tumors of the Central Nervous System) [1] is the most frequent primary brain tumor with very poor survival rate (median of 14.6 months) [2]. Currently, treatment bases on surgical resection if feasible, followed by radiotherapy and/or oral chemotherapy with temozolomide (TMZ) – alkylating agent inducing toxic DNA lesions like O6-methylguanine, N7-methylguanine or N3-methylalanine. These, become highly lethal for cancer cells when DNA repair systems are disrupted [3, 4].

One of the hallmarks of the cancerous cells is genomic instability responsible for accumulation of further genome rearrangements and tumor progression [5]. Development of such abnormalities in primary DNA repair systems results in activation of compensatory DNA repair mechanism, ipso facto, inducing cell “addiction” to the changes it carries. It has been suggested that cancer-specific abnormalities in the functioning of DNA repair systems and pathway redirection events might be responsible for the resistance and survival of cancer cells after exposure to genotoxic stress [6].

DNA double-strand breaks (DSBs) are the most toxic among DNA lesions and can be responsible for genome rearrangements leading to genomic instability, neoplastic transformation and cell death [7]. DSBs can arise due to the exposure to ionizing radiation (IR), reactive oxygen species (ROS) or genotoxic drugs [8]. Two mechanisms predominantly responsible for repair of DSBs in proliferating cells are BRCA1/2-mediated homologous recombination (HR) and DNA-PK-mediated non-homologous end-joining (D-NHEJ). When proper functioning of one of these pathways is compromised, cells redirect functions to an alternative mechanism – PARP1-dependant backup NHEJ (B-NHEJ) [9–11]. PARP1 is a protein playing a critical role in other processes decreasing the number of lethal DSBs - by activation of base excision repair (BER), single strand break (SSB) repair or HR activation at stalled replication forks [12–13]. PARP inhibitors are currently used in synthetic lethality-based personalized therapy in patients with breast and ovarian cancer deficient in BRCA1/2-mediated HR [14–15]. We hypothesized that deficiencies in DSB repair pathways could sensitize glioblastoma cells to PARP inhibitor (PARPi) BMN673 especially when combined with DSB-inducing drug temozolomide [16–18].

## RESULTS

### Expression of genes involved in DSB repair in normal human astrocytes and glioblastoma cells

**Figure 1:**
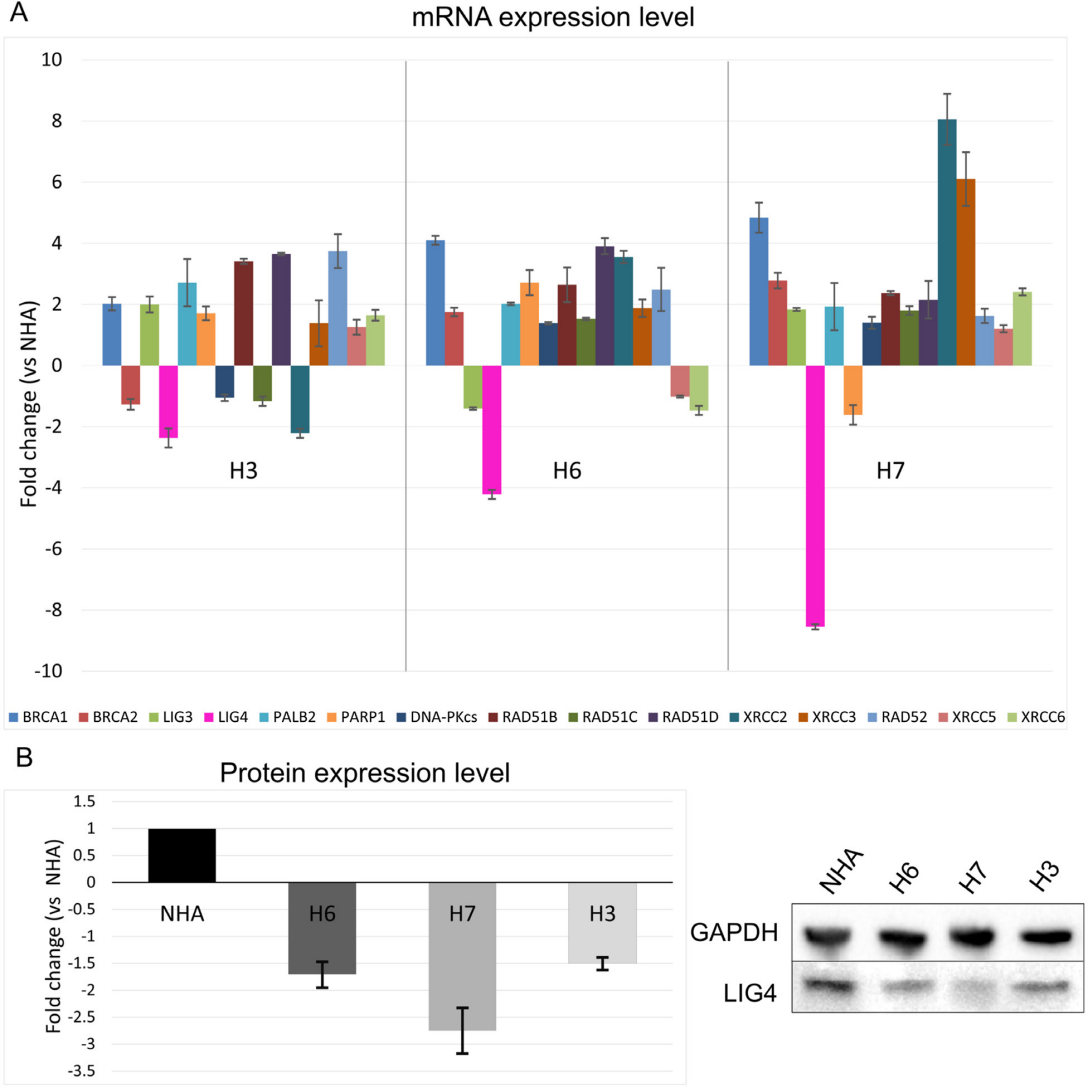
Expression profiles of genes involved in HR, D-NHEJ and B-NHEJ repair systems in glioblastoma cells vs normal human astrocytes (**A**) mRNA expression level of 15 indicated genes in primary human glioblastoma cell lines (H3, H6 and H7) was normalized to the expression of reference gene – 18S rRNA. Data are presented as a fold change in reference to normal human astrocytes (NHA). Results represent mean value ± SD from 3 independent experiments each performed in triplicates. (**B**) Protein expression level presented as fold change in comparison to NHA where expression was set as 1. The expression level was normalized to the reference protein, GAPDH. Mean ± SD was calculated form 3 independent experiments. Representative Western protein expression analysis of LIG4 and GAPDH (loading control) is shown.

In order to utilize personalized synthetic lethality approach we determined the expression profile for 3 patient-derived glioblastoma primary cell lines and compared it to the profile of normal human astrocytes (NHA). The subject of our interest were 15 genes involved in DSB repair pathways (BRCA1, BRCA2, PALB2, RAD51B, RAD51C, RAD51D, XRCC2, XRCC3, RAD52 taking part in HR; LIG4, DNA-PKcs, XRCC5, XRCC6 in D-NHEJ; and PARP1, LIG3 in B-NHEJ). Significant changes in the mRNA expression profile of LIG4 was found between glioblastoma cell lines and NHA (Figure 1A). Decreased level of LIG4 at the protein level was then confirmed by Western blot in cancer cell lines in comparison to normal astrocytes (Figure 1B). For further experiments H6 and H7 primary cell lines were chosen due to abundant downregulation of LIG4.

### Patient-derived glioblastoma cell lines carry tumor-unique molecular markers

**Figure 2:**
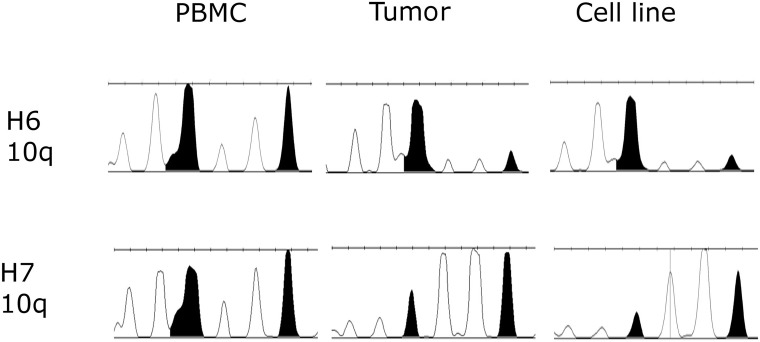
Loss of heterozygosity (LOH) examined in two primary tumor samples and established primary cell lines H6 and H7 in comparison to normal corresponding blood samples LOH in 10q locus was been observed in all neoplastic samples. As expected, tumor tissue expressed lower level of LOH due to the presence of both normal and cancer cells.

Loss of heterozygosity (LOH) in chromosome loci 10q, 10p and 22q were reported to be one of the most commonly occurring abnormalities during astrocytoma progression [19–20]. Therefore, we analyzed LOH in these locations in order to confirm the presence of glioblastoma cells in tumor samples and established primary cell lines H6 and H7. LOH was examined in two resected primary tumors and two corresponding primary cell lines in comparison to blood samples. LOH in 10q locus were detected in all neoplastic samples (Figure 2) whereas no aberrations were noted in 10p and 22q locus. The presence of the wild-type DNA in the tumor samples have been observed, which may refer to the heterogeneity of cell population in the tumor bulk. Nonetheless control vs corresponding tumor peak height ratio was lower than 50%, whereas in cell lines was more than 65–70%, strongly suggesting that 10q aberration has been propagated in cell culture condition.

### Response of patient-derived glioblastoma cells and normal astrocytes to PARP1 inhibitor used alone and in combination with alkylating agent

**Figure 3:**
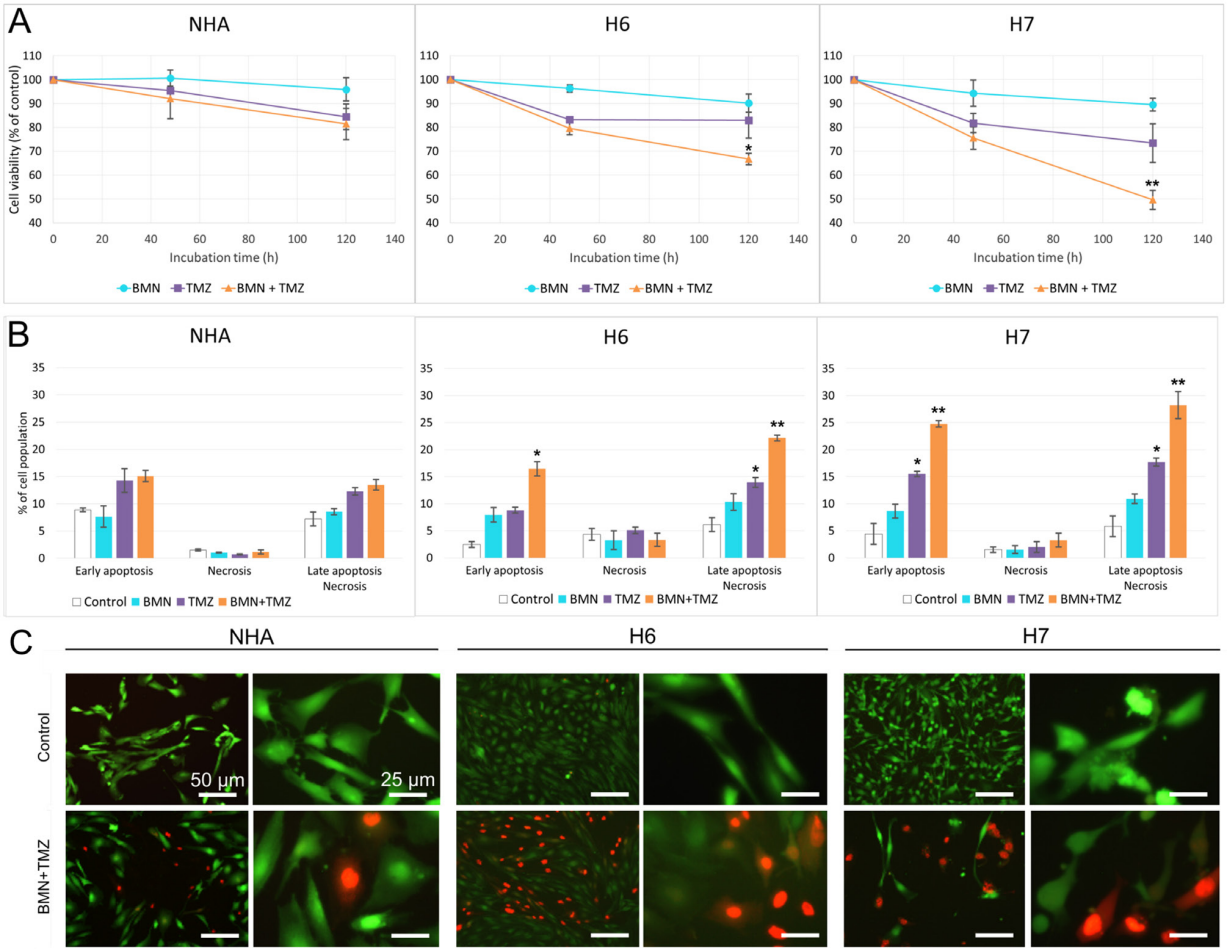
BMN673+TMZ anti-glioblastoma effect H6, H7 and NHA cells were treated with BMN673 (BMN) and/or TMZ. (**A**) Viability measured as population of Annexin V/PI negative cells in comparison to vehicle-treated control after 48 h and 120 h. Results represent mean % ± SD of 3 independent experiments, ^*^p < 0.05, ^**^p < 0.001 in comparison with control. (**B**) Quantitative representation of flow cytometry results after 120 h of treatment. Results represent mean value ± SD from 3 independent repeats, ^*^p < 0.05, ^**^p < 0.001 in comparison with control. (**C**) Morphological changes of normal and cancer cells after 120 h of treatment with BMN673 + TMZ or vehicle (Control). Cells were stained with Calcein AM/ propidium iodide. Note the typical morphological features of cell death: loss of structural framework of nuclei, condensation of chromatin, cell shrinkage and nuclear fragmentation (observed mostly in higher magnification). Cells were analyzed under an inverted fluorescence microscope (Olympus IX70), magnification x100 (scale bar = 50 μm) and x400 (scale bar = 25 μm).

To analyze the potential anti-glioblastoma effect of PARP inhibitor (BMN673) used either alone or in combination with alkylating agent (TMZ), double staining with propidium iodide (PI) and annexin V was used. Annexin V staining in conjunction with vital dye (PI) distinguishes viable cells from dead, and also early apoptotic cells from necrotic cells. When compared to individual agents, the combination of BMN673 + TMZ exerted significantly stronger anti H6 and H7 glioblastomas effect with only minimal toxicity to normal astrocytes (Figure 3A). The flow cytometry result indicates also that post-treatment cell death occurs majorly via apoptosis as cells were getting accumulated in Q4 quadrant (Annexin V^+^, PI^-^) and then shifting to Q2 quadrant (Annexin V^+^, PI^+^) what would characterize slow externalization of phosphatidylserine and prolonged annexin V binding which is typical for apoptosis (Figure 3B) (Supplementary Figure 1). The results were also confirmed with trypan blue staining (Supplementary Figure 2).

Morphological changes induced by BMN673 +/- TMZ were assessed by Calcein AM/PI double staining (Figure 3C). Cells treated with the inhibitors showed the characteristic hallmarks of cellular homeostasis disorders (cellular membrane damage, cell shrinkage and their fragmentation). These morphology changes were much more noticeable in cancer than in normal cells. These alterations of cellular morphology were in agreement with the increasing number of dead cells stained with PI especially in samples treated with BMN673 + TMZ.

**Figure 4:**
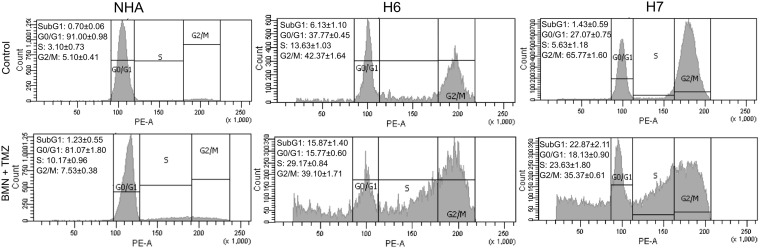
Cell cycle distribution of H6 and H7 glioblastoma cells and NHA cells treated or not with BMN673 + TMZ Representative graphs of normal human astrocytes (NHA) and H6 and H7 primary cell lines after 120 h incubation with the drugs (BMN + TMZ) or vehicle (Control). Left upper corner of each variant includes quantitative representation of cell population in each cell cycle phase – SubG1, G0/G1, S, G2/M. Values represent mean ± SD from 3 independent experiments.

The impact of BMN673 + TMZ on cell cycle phase distribution of glioblastoma cells and normal human astrocytes was analyzed by flow cytometry (Figure 4). The effect of drugs was visible in H6 and H7 glioblastomas as elevation of SubG1 and S phase populations but a picture typical for G2/M arrest was not detected. Interestingly, drug-induced changes in cell cycle phases for NHA cells were slight to none.

**Figure 5:**
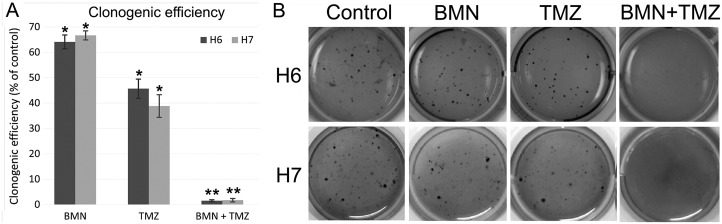
Clonogenic potential of patient-derived glioblastomas after treatment with BMN673 and/or TMZ (**A**) Cells were treated with either vehicle, BMN673, TMZ and BMN673 + TMZ followed by soft agar culture for 2–3 weeks. Clonogenic efficiency is shown as mean ± SD % of control (cells treated with vehicle) from 3 independent experiments, ^*^p < 0.05 and ^**^p < 0.001 in comparison to control. (**B**) Photographs of a representative experiment.

Clonogenic assay was used to test the impact of drugs on colony formation ability of cancer cells. When used alone, only TMZ had a significant influence on long-term clonogenic efficiency whereas BMN673 + TMZ were able to almost completely abrogate clonogenic ability of LIG4-deficient glioblastoma cells (Figure 5).

### Combination of BMN673 and TMZ induces accumulation of toxic DSBs in patient-derived glioblastoma cells

**Figure 6:**
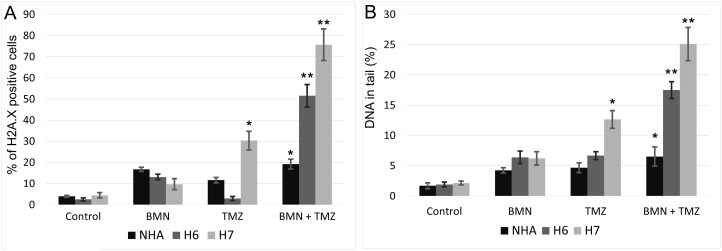
Accumulation of DSB in BMN673+TMZ-treated H6 and H7 glioblastoma cells and in NHA cells (**A**) DSBs were detected by γH2A.X immunofluorescence. Bars show mean percentage of γH2A.X –positive cells ± SD from 3 independent experiments. (**B**) DSBs were detected by neutral comet assay. Bars show mean percentage of DNA in tail ± SD from 50 randomly selected cells in 3 independent experiments. ^*^p < 0.05 and ^**^p < 0.001 when compared to control.

Phosphorylation of serine 139 on histone 2A.X (γH2A.X) can be used as a marker of DSBs [21]. TMZ treatment increased γH2A.X immunofluorescence in H7 primary cell line (Figure 6A). This effect was remarkably enhanced in both H6 and H7 cell lines when BMN673 and TMZ were used in combination. In NHA cells the level of γH2A.X positive cells stayed at relatively low level regardless from the treatment used.

Neutral comet assay was also employed to detect DSBs after treatment with BMN673 and/or TMZ. After treatment with individual drugs only TMZ enhanced the percentage of DNA in tails of H7 cells in comparison to NHA cells (Figure 6B). Combination of BMN673 and TMZ caused significant increase of DSBs in both glioblastoma cell lines.

### Rescue of LIG4 expression caused resistance to BMN673 + TMZ treatment

**Figure 7:**
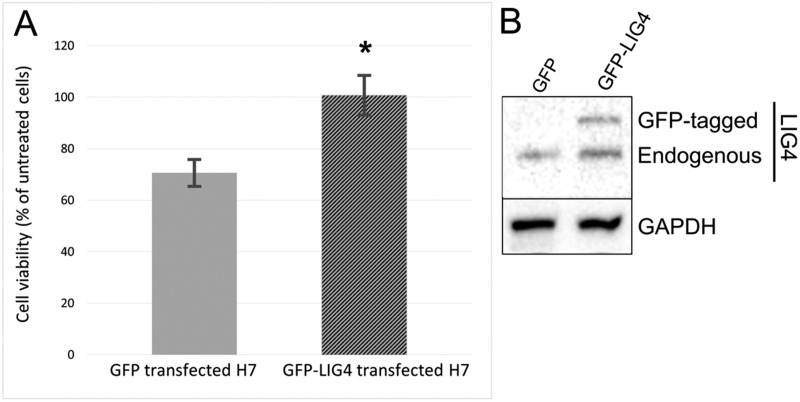
Sensitivity to BMN673+TMZ depends on LIG4 expression (**A**) The effect of BMN673 in combination with TMZ on H7 cells transfected with GFP (Control) or GFP-LIG4 expression plasmid. Results represent mean % of viable cells ± SD from 3 independent experiments, ^*^p < 0.05 comparing with Control. (**B**) Representative Western blot of LIG4 expression in H7 cells transfected with control GFP plasmid and with vector carrying GFP-LIG4. GAPDH is included as a loading control.

To determine the role of reduced expression of LIG4 in sensitivity of glioblastoma cells to BMN673+TMZ, H7 cells were transfected with the plasmid carrying LIG4 cDNA followed by treatment with the drugs. Elevated expression of LIG4 resulted in resistance of H7 glioblastoma primary cell line to BMN673 + TMZ (Figure 7).

## DISCUSSION

Due to the growing knowledge of genetic and epigenetic changes in tumors the concept of synthetic lethality became lately one of the main areas of searching for new therapy candidates. The phenomenon occurs when simultaneous loss of two genes causes cell death whereas loss of each of these genes individually is not lethal [11]. For instance, BRCA1/2 deficient tumors with impaired homologous recombination repair were reported to be sensitive to PARP inhibition [16–17]. PARPi could be used in combination with the agents inducing DNA damage like doxorubicin, radiation or alkylating drugs [22]. Therefore, we postulated that combination of PARPi may significantly improve the therapeutic outcome of currently used TMZ-based therapy and specifically eradicate glioblastoma cells with disrupted DSB repairing pathways.

LIG4 is a crucial element of D-NHEJ pathway and its low level might result in ineffective functioning of this repair system. Downregulation in LIG4 was previously described in patient-derived high-risk neuroblastomas and correlated with higher stage of disease and lower survival probability [23]. Analysis of available mRNA gene expression databases revealed cohorts of glioblastomas displaying lower expression of LIG4. Mechanisms responsible for reduced expression of LIG4 are not known, but our recent report suggested that inefficient JAK2-STAT5 and/or PI3K-AKT pathways may play a role [24].

To examine the potential therapeutic aspect we generated primary glioblastoma cells with downregulated LIG4 when compared to normal human astrocytes. PARPi BMN673 in combination with alkylating agent TMZ was effective against patient-derived glioblastoma cells displaying downregulation of LIG4 but not against normal human astrocytes. Downregulation of LIG4 in glioblastoma cells was directly responsible for enhanced sensitivity to BNM673 as restoration of LIG4 expression resulted in resistance to the treatment.

We postulate that D-NHEJ deficiency resulting from downregulation of LIG4 could be synthetically lethal with B-NHEJ deficiency induced by PARPi in glioblastoma cells exposed to TMZ-induced DNA damage. In concordance, we demonstrated that LIG4 deficient melanoma cells were highly sensitive to the combination of an alkylating agent dacarbazine and PARPi [18]. Morevoer, HCT116 *Lig4-/-* cells were sensitive to the combination of PARPi with radiotherapy [25].

**Figure 8:**
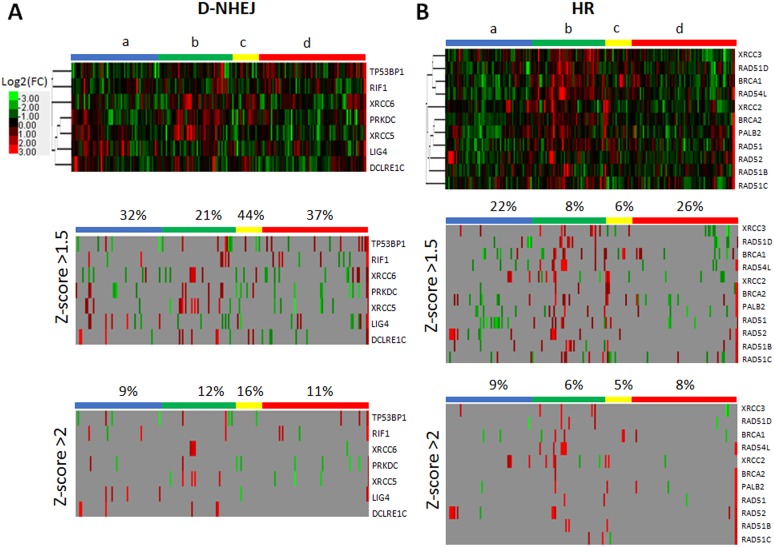
Microarray-based gene expression profiling for the genes in (A) D-NHEJ pathway and (B) HR pathway Data was obtained for 191 glioblastoma patients manifesting the following phenotypes: a – proneural (*n* = 56); b – proliferative (*n* = 48); c- proliferative-mesenchymal (*n* = 18); d – mesenchymal (*n* = 69). Percent above column bar represents number of samples with at least one downregulated gene within the phenotype group.

Although downregulation/mutation of LIG4 (and its partner XRCC4) was detected only in approximately 4% of glioblastomas in The Cancer Genome Atlas (TCGA) database [26] inhibition/inactivating mutation of other members of D-NHEJ potentially impairing DSB repair activity were present in up to 20% of the cases. Moreover, transcriptome analysis by microarrays detected downregulation of at least one member of D-NHEJ pathway (including LIG4) in 191 glioblastomas manifesting the proneural, proliferative, proliferative-mesenchymal and mesenchymal phenotypes (Figure 8A) [27]. In addition, multiple glioblastoma samples displayed downregulation of at least one gene in HR pathway (Figure 8B) suggesting their sensitivity to synthetic lethality triggered by PARPi [28].

In summary, this study implicates potential therapeutic effect of PARPi used in combination with DNA-damaging agents in D-NHEJ-deprived glioblastoma cells. Therefore patient pre-selection based on expression of DNA repair genes may be applied for personalized medicine approach to improve the effectiveness of anti-glioblastoma therapy.

## MATERIALS AND METHODS

### *In vitro* cell culture

Glioblastoma specimens, histopathologically classified as clinical stage IV, were obtained from patients of Department of Neurosurgery, Surgery of Spine and Peripheral Nerves, Medical University of Lodz (University Hospital WAM-CSW Łódź) and Department of Neurosurgery, Medical University of Lodz (Copernicus Memorial Hospital, Łódź, Poland). Cell cultures derived from specimens were established in the Laboratory of Molecular Genetics, University of Lodz and named H3, H6 and H7. After several washes tissue fragments were minced with scalpel and cells were filtered through 70 μM pore size cell strainer. Glioblastoma cells were cultured in DMEM medium (Lonza, Basel, Switzerland) supplemented with 10% FBS (Lonza), 100 IU/ml penicillin, 100 μg/ml streptomycin (Lonza) and gentamycin 50 μg/ml (Lonza) in a humidified atmosphere containing 5% CO_2_ at 37°C. The normal human astrocytes NHA (Lonza) were grown in ABM Basal Medium supplemented with AGM BulletKit (Lonza) and cultured according to the protocol provided by manufacturer.

### Isolation of DNA from tumor, blood and cell culture samples and loss of heterozygosity analysis

Loss of heterozygosity (LOH) analysis was performed using microsatellite markers. The aim of the experiment was to verify presence of genetic aberrations specific for glioblastoma cells in suspected cancerous tissue. The samples were examined for LOH using sets of DNA samples isolated from tumor bulk specimen and corresponding cell culture and peripheral blood samples (PBMC). Isolation and purification of genomic DNA was performed with Genomic Mini and Blood Mini isolation kits (A&A Biotechnology) according to the manufacturer's protocol. The microsatellite markers D10S1709 (10q) (F–GTGAGTCCAGAATCACCCC, R–CAGTGGAAATGGCTCATTTG), D10S1172 (10p) (F–GGATACTACCAAGAGAGAG, R– ATCATCTATCTCTACTATCTG), D22S283 (22q) (F–ACC AACCAGCATCATCAT, R–AGCTCGGGACTTTCTGAG) were selected using the NCBI database [20, 21]. The F primers were 5’-labelled with Fam fluorochrome (Sigma). Each reaction was amplified in volume of 25 μl containing 50 ng of DNA template, dNTP, KAPA Taq DNA Polymerase (Kapa Biosystems) and forward/reverse primers. PCR reaction was carried as follows: 95°C 3 min, (95°C 30s, temperature depending on primer pair 30s, 72°C 45s)x32, 72°C 4 min. PCR products were visualized with a 16-capillary electrophoresis 3130xl Genetic Analyzer (Applied Biosystems). The analysis was performed using Gene mapper 4.1 software and verified manually. Loss of heterozygosity was judged to be present if the allelic signal intensity of the tumor sample was reduced by at least 50% relative to the corresponding allele in the patient's control DNA (PBMC).

### RNA isolation, reverse transcription and Real-Time PCR

RNA isolation and purification was performed using RNA isolation kit (A&A Biotechnology). In the next step samples were transcribed into cDNA with SuperScript II Reverse Transcriptase (Invitrogen, Life Technologies, Carlsbad, California, USA) according to the manufacturer's protocol. Real-Time PCR quantitation was carried out using TaqMan Real-Time PCR Master Mix and TaqMan probes (Applied Biosystems, Life Technologies, Carlsbad, California, USA) detecting genes which products are involved in DSB repair pathways (BRCA1, BRCA2, LIG3, LIG4, PALB2, PARP1, PRKDC, RAD51B, RAD51C, RAD51D, XRCC2, XRCC3, RAD52, XRCC6, XRCC7). 18S rRNA TaqMan probe was included as the reference gene. The parameters for Agilent Technologies Stratagene Mx300SP instrument were 95°C for 10 minutes, 30 cycles of 95°C for 15 seconds and 60°C for 60 seconds.

### Protein isolation and western blot analysis

Protein extraction was performed incubating cell pellet with the mixture of RIPA buffer (Sigma) and protease inhibitor cocktail (Thermo Scientific, Rockford, Illinois, USA). After concentration measurement, 30 μg of cell lysates was resolved on 4–20% ExpressPluS PAGE Gel (GenScript, Piscataway, New Jersey, USA). The proteins were then transferred onto PVDF Transfer Membrane (Thermo Scientific, Rockford, Illinois, USA) using eBlot Protein Transfer device (GenScript, Piscataway, New Jersey, USA). Membranes were blocked and blotted overnight with primary antibodies recognizing LIG4 and GAPDH (Santa Cruz Biotechnology, Dallas, Texas, USA). Membranes were then washed and incubated 1 h with secondary anti-mouse antibody conjugated with HRP (Cell Signaling Technology, Danvers, Massachusetts, USA). The result was visualized using Pierce ECL Western Blotting Substrate (Thermo Scientific, Rockford, Illinois, USA) and BioRad Universal Hood II with Chemiluminescence System (BioRad, Hercules, California, USA).

### Drug treatment

Normal astrocytes and glioblastoma cells were plated in a 6-well plate at a density of 2 × 10^5^ viable cells per well. Cells were cultured with 50 nM BMN673 (Selleckchem), 6.25 μM TMZ (Sigma Aldrich), BMN673 + temozolomide or vehicle for 48 h followed by the second dose of the compounds and another 72 h of incubation.

### Calcein AM/propidium iodide double staining

After the indicated treatments, normal and cancer cells were incubated for 30 min at 37°C with the mixture of 2 mM Calcein AM and propidium iodide 1 mM (Life Technologies, USA) diluted in PBS. Fluorescence emitted by stained cells was then observed in an inverted fluorescence microscope (Olympus IX70, Japan).

### Flow cytometry

Flow cytometry and staining with propidium iodide and FITC Annexin V (BD Biosciences) was used to assess changes in viability and to track the mechanism of cell death after treatment. Cells were prepared and analyzed according to the FITC Annexin Apoptosis Detection Kit II (BD Biosciences). To analyze the influence of the compounds on glioblastoma and NHA distribution in cell cycle, cells fixed with 70% cold ethanol were stained with propidium iodide with addition of RNase (BD Biosciences) and analyzed. The extent of DNA DSBs measured by phosphorylation of H2A.X histone was obtained using Alexa Fluor 647 Mouse Anti-H2A.X (pS139) antibody (Becton Dickinson, San Jose, California, USA) after 48 h treatment with the compounds. Fixed cells were washed resuspended in 20 μl BD Perm/Wash™ buffer and stained for 20 min with H2A.X antibody (5 μl/test). All the experiments were performed using a FACS Canto II cytometer (Becton Dickinson, San Jose, California, USA).

### Neutral comet assay

Neutral comet assay was performed according to the protocol used in the previous research [19] on cells cultured for 48 h with either drugs or vehicle. Fifty comet images were randomly selected for each treatment variant and the percentage of DNA in the tail (% tail DNA) was measured. The mean value for this parameter was taken as an index of DSBs in the given sample.

### Clonogenic assay

To examine clonogenic activity glioblastoma cells were first cultured with drugs or vehicle for 48 h followed by the second dose of the compounds and another 72 h of incubation. After treatment cell viability was determined by staining with trypan blue and 10^3^ cells were resuspended in 700 μl of soft agar (DMEM, 0.4% w/v) and plated over 700 μl of solidified agar underlay (DMEM, 0,5% agar) on a 12-well plate. After solidifying cell layer was covered with medium (changed weekly). After 2–3 weeks colonies were stained with crystal violet (0.5% w/v) and counted under the microscope. Clonogenic efficiency was expressed as percent of untreated control (no. of colonies after treatment vs no. of colonies in control sample × 100%).

### Ectopic expression of LIG4

Glioblastoma H6 cells were transfected with pCMV6-AC-GFP plasmid containing human LIG4 cDNA (OriGene Technologies). The method was performed using Lipofectamine 2000 (Invitrogen, Life Technologies, Carlsbad, California, USA). GFP-positive cells were sorted 48 h after transfection.

### Microarrays

Microarray data sets were obtained from NCBI GEO (GSE13041). Gene expression profiling was performed as described before [29–30]. Microarray was subset for D-NHEJ genes and HR genes. Z-score cutoffs were set at 1.5 and 2 to detect upregulated and downregulated genes as described before [18].

### Statistical analysis

Data was accessed in three independent experiments and presented as mean ± SD. Results were compared using two tailed Student *t* test. *P* values lower than 0.05 were considered significant. The synergistic effect of drugs was studied using response additivity approach.

### Study approval

Studies performed on cells derived from surgical specimens were approved by the Ethical Commission of the Medical University of Lodz (no. RNN/194/12/KE) and informed consent was obtained from all patients.

## SUPPLEMENTARY MATERIALS FIGURES



## Abbreviations

BER: base excision repair
DSB: DNA double strand break
HR: homologous recombination
IR: ionizing radiation
LOH: loss of heterozygosity
NHA: normal human astrocytes
NHEJ: non-homologous end-joining
PBMC: peripheral blood mononuclear cells
PI: propidium iodide
ROS: reactive oxygen species
SD: standard deviation
SSB: DNA single strand break
TMZ: temozolomide
