# Supplementary material for: Eradication of LIG4-deficient glioblastoma cells by the combination of PARP inhibitor and alkylating agent
Source: Oncotarget. 2018 Dec 7;9(96):36867–77. doi: 10.18632/oncotarget.26409 (PMC6305145; doi:10.18632/oncotarget.26409)
Supplement: Supplementary file 1 [file oncotarget-09-36867-s001.pdf]

Eradication of LIG4-deficient glioblastoma cells by the combination of PARP inhibitor and alkylating agent

SUPPLEMENTARY MATERIALS

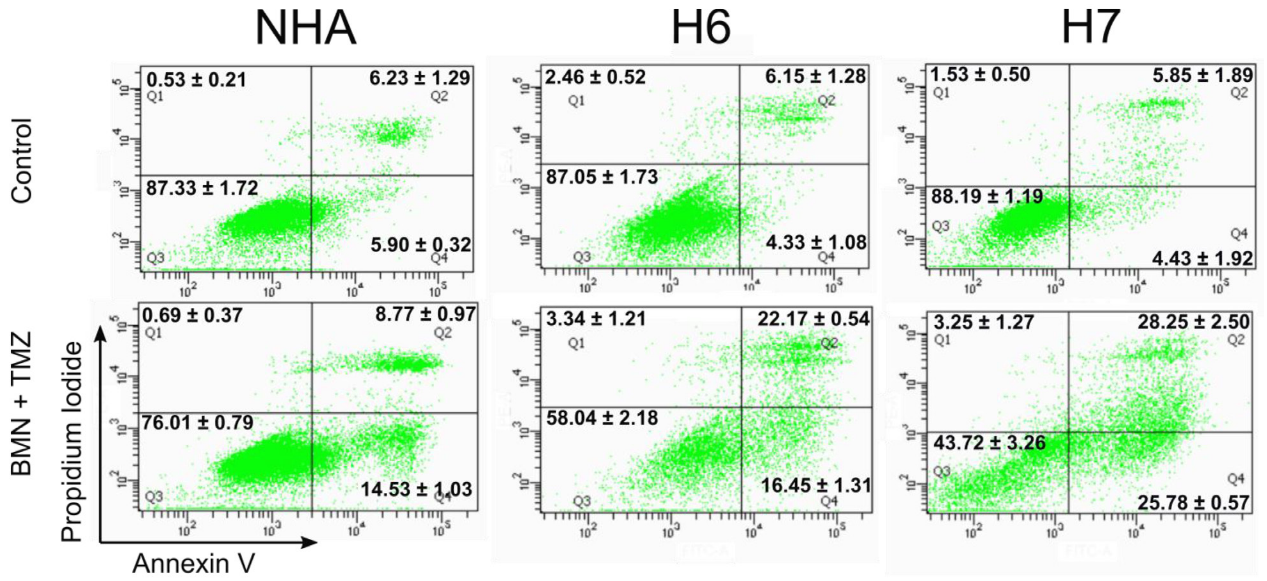

Supplementary Figure 1: Flow cytometry dot-plots representing results of double staining with Annexin V and PI. Results represent mean ± SD of 3 experiments. Q1 quadrant represents necrotic PI-positive only cells (Annexin V^{-}, PI^{+}), Q2 – late apoptotic and necrotic (Annexin^{+}, PI^{+}), Q3 – viable cells (AnnexinV^{-}, PI^{-}) and Q4 – early apoptotic cells (AnnexinV^{+}, PI^{-}).

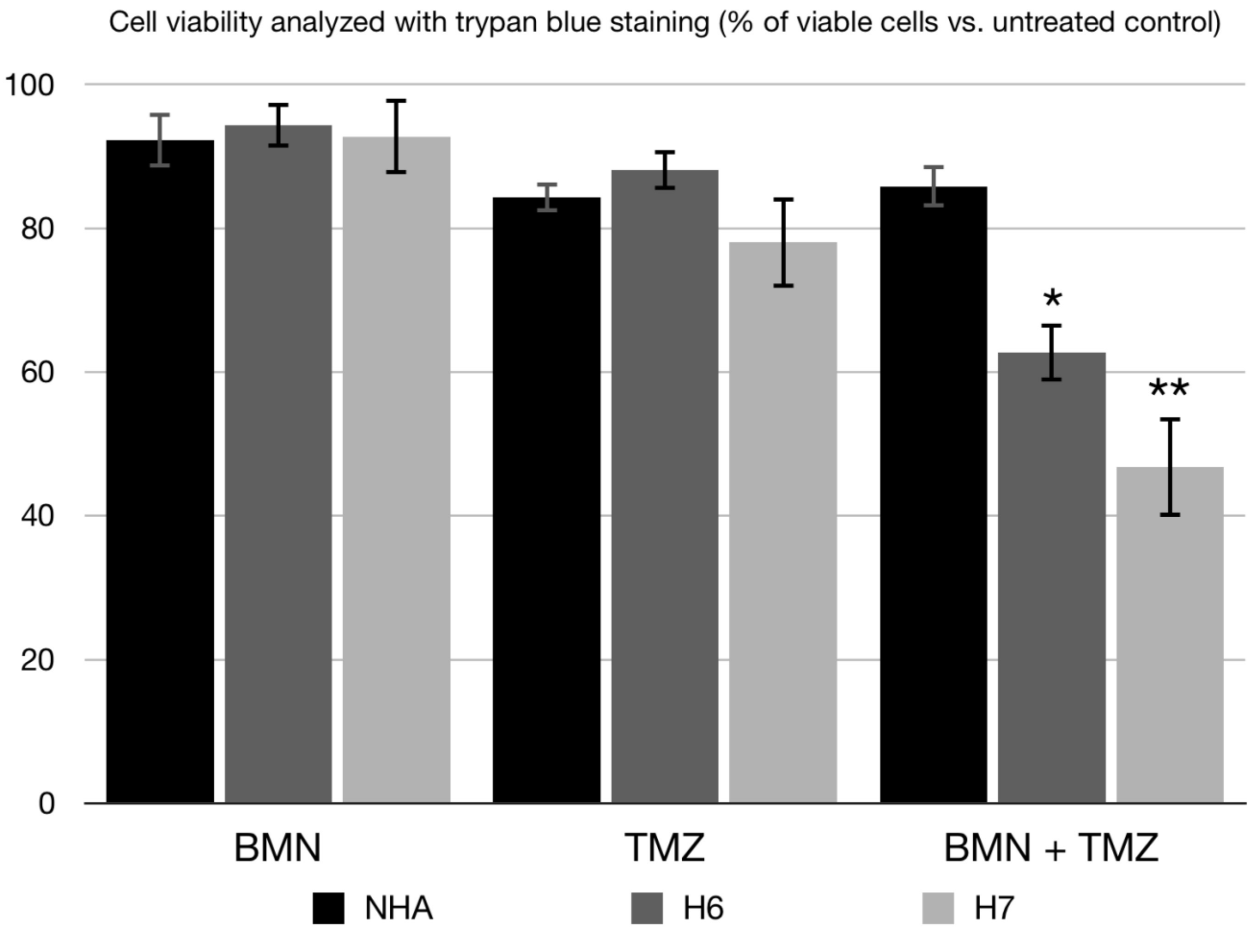

Supplementary Figure 2: Cell viability counted after trypan blue staining. Cells were incubated with either vehicle, BMN673 (50 nM), TMZ (6.25 μM) or BMN673 + TMZ for 120 h. Results represent mean ± SD of 3 experiments. $^{*}p < 0.05$, $^{**}p < 0.001$ in comparison to control.
